# Circulating Trends of Influenza and Other Seasonal Respiratory Viruses among the US Department of Defense Personnel in the United States: Impact of the COVID-19 Pandemic

**DOI:** 10.3390/ijerph19105942

**Published:** 2022-05-13

**Authors:** Wenping Hu, Anthony C. Fries, Laurie S. DeMarcus, Jeffery W. Thervil, Bismark Kwaah, Kayla N. Brown, Paul A. Sjoberg, Anthony S. Robbins

**Affiliations:** 1 The Department of Defense Global Emerging Infections Surveillance Branch, Armed Forces Health Surveillance Division, Wright-Patterson Air Force Base, Dayton, OH 45433, USA; laurie.demarcus.ctr@us.af.mil (L.S.D.); jeffrey.thervil.ctr@us.af.mil (J.W.T.); bismark.kwaah.ctr@us.af.mil (B.K.); kayla.brown.16.ctr@us.af.mil (K.N.B.); paul.sjoberg.1.ctr@us.af.mil (P.A.S.); anthony.robbins.5@us.af.mil (A.S.R.); 2 JYG Innovations LLC, Dayton, OH 45414, USA; 3 U.S. Air Force School of Aerospace Medicine, Wright-Patterson Air Force Base, Dayton, OH 45433, USA; anthony.fries.1@us.af.mil

**Keywords:** influenza, respiratory virus, SARS-CoV-2, surveillance, pandemic

## Abstract

The objective of this study was to evaluate the impact of the COVID-19 pandemic on the circulation of influenza and other seasonal respiratory viruses in the United States. All data were obtained from the US Department of Defense Global Respiratory Pathogen Surveillance Program over five consecutive respiratory seasons from 2016–2017 through to 2020–2021. A total of 62,476 specimens were tested for seasonal respiratory viruses. The circulating patterns of seasonal respiratory viruses have been greatly altered during the pandemic. The 2019–2020 influenza season terminated earlier compared to the pre-pandemic seasons, and the 2020–2021 influenza season did not occur. Moreover, weekly test positivity rates dramatically decreased for most of the seasonal respiratory viruses from the start of the pandemic through spring 2021. After the easing of non-pharmaceutical interventions (NPIs), circulations of seasonal coronavirus, parainfluenza, and respiratory syncytial virus have returned since spring 2021. High rhinovirus/enterovirus activity was evident throughout the 2020–2021 respiratory season. The findings suggest a strong association between the remarkably changed activity of seasonal respiratory viruses and the implementation of NPIs during the COVID-19 pandemic. The NPIs may serve as an effective public health tool to reduce transmissions of seasonal respiratory viruses.

## 1. Introduction

Severe Acute Respiratory Syndrome Coronavirus 2 (SARS-CoV-2) spreads globally, resulting in an unprecedented public health crisis [1]. As of the end of the 2020–2021 respiratory season, it has been over one and a half years since the World Health Organization declared the COVID-19 pandemic in March 2020. During this time, policy makers worldwide have implemented stringent non-pharmaceutical interventions (NPIs) strategies such as lockdown, travel restrictions, and personal/social protective measures, in order to reduce the transmission of SARS-CoV-2 [2,3]. Meanwhile, the implementation of such public health measures appears to have exerted great impacts on the incidence of seasonal respiratory viruses. Studies conducted in both Northern [4,5,6,7] and Southern hemispheres [8,9,10,11] demonstrated that the percentage of infection with seasonal respiratory viruses dramatically decreased during much of 2020 and early 2021. Moreover, delayed activity of the respiratory syncytial virus (RSV) was observed [12,13]. With increasing uptake of vaccination against SARS-CoV-2, NPIs have been relaxed to varying extents in different parts of the United States since March 2021. It is unknown whether the seasonal respiratory viruses would continue to be markedly decreased in the late phase of the 2020–2021 season, and further into the next respiratory season. Year-round respiratory virus surveillance is of clinical importance [14]. Therefore, it is crucial to continue to closely monitor the circulation of influenza and other seasonal respiratory viruses, and determine how the ongoing pandemic may affect the transmission patterns of those viruses.

Every year, the Department of Defense Global Respiratory Pathogen Surveillance (DoDGRS) program performs routine respiratory pathogen surveillance among the Department of Defense’s service members and their beneficiaries. The objective of this study was based on the DoDGRS data collected over five consecutive influenza seasons (2016–2021), to characterize circulating trends of influenza and other seasonal respiratory viruses among the Department of Defense personnel in the United States during the COVID-19 pandemic, and to compare the trends with those that occurred in the pre-pandemic period.

## 2. Methods

### 2.1. Surveillance Data

We obtained respiratory pathogen surveillance data on US Department of Defense personnel from the DoDGRS program over five consecutive respiratory seasons from 2016–2017 to 2020–2021. All patients seeking outpatient medical care were selected at sentinel or participating sites throughout the United States, using criteria which meet an influenza-like illness (ILI) case definition. ILI is defined as a patient presenting with (1) a fever (≥38 °C) and a cough or sore throat within 72 h after illness onset, or (2) symptoms determined by a physician to be an ILI case. Respiratory specimens were collected from ILI patients by nasopharyngeal wash or nasopharyngeal swab, and tested via a multiplex respiratory pathogen panel. In addition, a reverse transcription polymerase chain reaction (RT-PCR) and/or viral culture were used to identify and confirm the influenza viruses and other seasonal respiratory pathogens. If the respiratory specimen tested positive for influenza virus, further testing was conducted to identify the influenza virus type and subtype/lineage. During the COVID-19 pandemic, patients who tested for both seasonal respiratory viruses and SARS-CoV-2 were not included for analysis, due to possible changes in test eligibility and testing procedure in detecting respiratory viruses.

### 2.2. Statistical Analysis

The weekly test positivity rate for each respiratory virus in a given respiratory season was defined as the percentage of the number of cases over the total number of tests for the epidemic week under surveillance. By using the same thresholds in the study of Feng et al. [15], the start of an influenza epidemic period was defined as the first week during which the weekly test positivity rate was >10% and remained >10% for at least two consecutive weeks. The end of an influenza epidemic period was defined as the last week during which the positivity rate was >10% and remained >10% for at least two consecutive weeks before decreasing to <10%. The duration of the influenza epidemic period was then calculated.

The four-week averages of test positivity rates of non-influenza seasonal respiratory viruses during the COVID-19 pandemic were compared with those in the pre-pandemic (average for three previous seasons, 2016–2017 through 2018–2019), using the GLM procedures of SAS (SAS Enterprise Guide 7.1, SAS Institute Inc., Cary, NC, USA) with the model

Y_i_ = μ + T_i_ + e_i_,

where μ = overall mean; T_i_ = effect of treatment (treatment was the COVID-19 pandemic and the pre-pandemic, i = 1, 2); and e_i_ = error term. A *p* < 0.05 was considered statistically significant.

## 3. Results

Influenza and other seasonal respiratory viruses detected over five respiratory seasons (2016–2017 through 2020–2021) are shown in Table 1. During the five respiratory seasons, a total of 62,476 tests for detection of seasonal respiratory viruses were performed; among which 13,536 (21.7%) were positive for influenza, 634 (1.0%) for adenovirus, 2189 (3.5%) for seasonal coronavirus, 1338 (2.1%) for human metapneumovirus, 1208 (1.9%) for parainfluenza, 1649 (2.6%) for RSV, and 6687 (10.7%) for rhinovirus/enterovirus. Of the total 13,536 influenza viruses detected, influenza A(H1N1)pdm09, influenza A(H3N2), and influenza B were 3769 (27.8%), 4624 (34.2%), and 3621 (26.8%), respectively, with the remaining being 1475 (10.9%) non-subtyped influenza A and 47 (0.3%) dual influenza viruses (Table 1).

**Figure 1:**
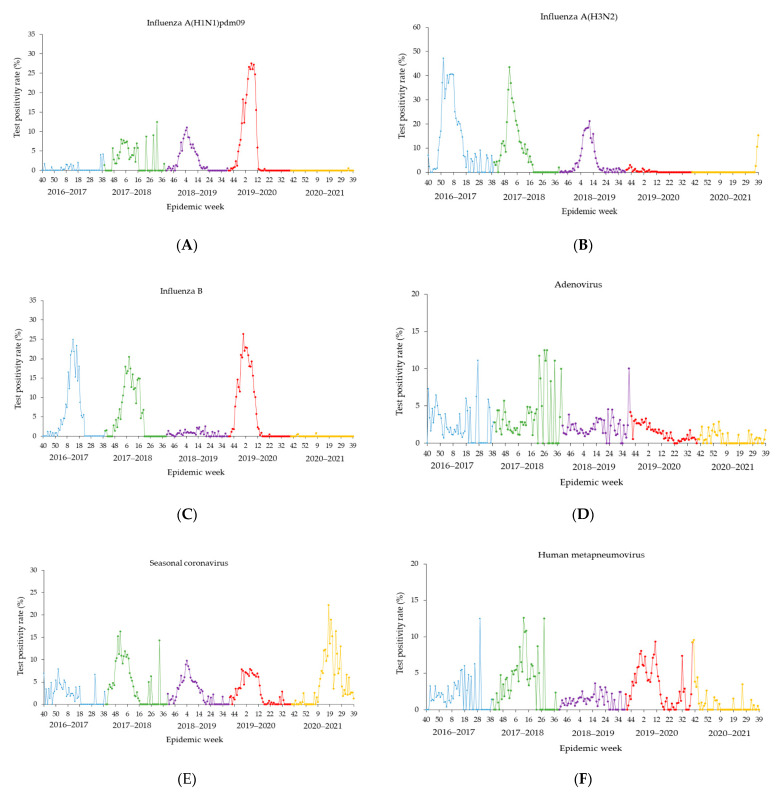

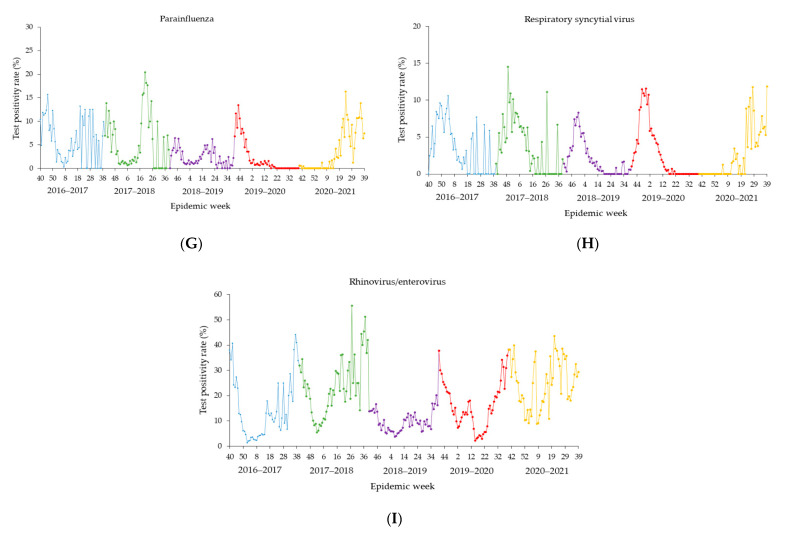
(**A**–**I**) Weekly test positivity rate of seasonal respiratory viruses over five respiratory seasons from 2016–2017 to 2020–2021. Epidemic week is the week of the epidemiologic year for which there is the National Notifiable Diseases Surveillance System (NNDSS) disease report, the United States.

**Figure 2:**
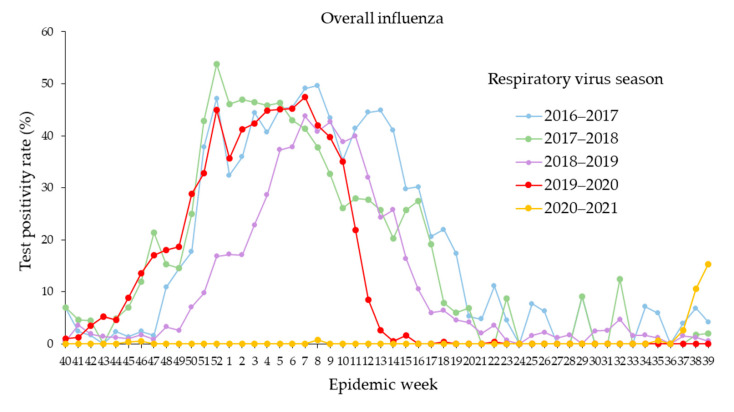
Seasonal activity of overall influenza including influenza A(H1N1)pdm09, influenza A(H3N2), and influenza B in respiratory seasons from 2016–2017 to 2020–2021. Epidemic week is the week of the epidemiologic year for which there is the National Notifiable Diseases Surveillance System (NNDSS) disease report, the United States. There are 53 weeks exceptionally in the 2020–2021 season, the positivity rate of overall influenza in week 53 was zero, and not shown in graph.

During three pre-pandemic influenza seasons (2016–2017 through 2018–2019), the influenza epidemic period started in as early as week 46 (18–24 November 2017) and ended in as late as week 19 (13–19 May 2017), lasting an average period of 21.7 weeks (range: 17–24 weeks) (Table 2). During the 2019–2020 respiratory season, influenza activity decreased rapidly after the United States declaration of the state of emergency in response to the COVID-19 pandemic on 13 March 2020 (Figure 1A,C and Figure 2). The influenza epidemic period of the 2019–2020 season ended in week 11 (14–20 March 2020), which was earlier compared to three previous influenza seasons (Table 2). Thereafter, the weekly positivity rates of influenza decreased from 8.4% in week 12 to 2.7% in week 13. The detection of influenza further decreased to near absence through the end of the 2019–2020 season. Moreover, the 2020–2021 influenza season did not occur. During the entire 2020–2021 respiratory season, there were a total of four influenza-positive cases including one influenza A(H1N1)pdm09 and three influenza B detected from 5518 specimens from the beginning through week 36. This was followed by increased influenza A (H3N2) circulation in the last three weeks 37–39 [5 (2.7%) in week 37; 20 (10.6%) in week 38; and 35 (15.4%) in week 39] (Figure 2).

**Figure 3:**
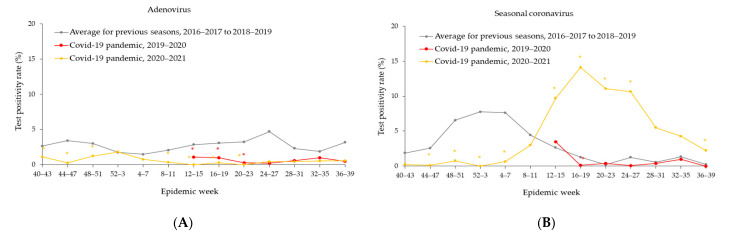

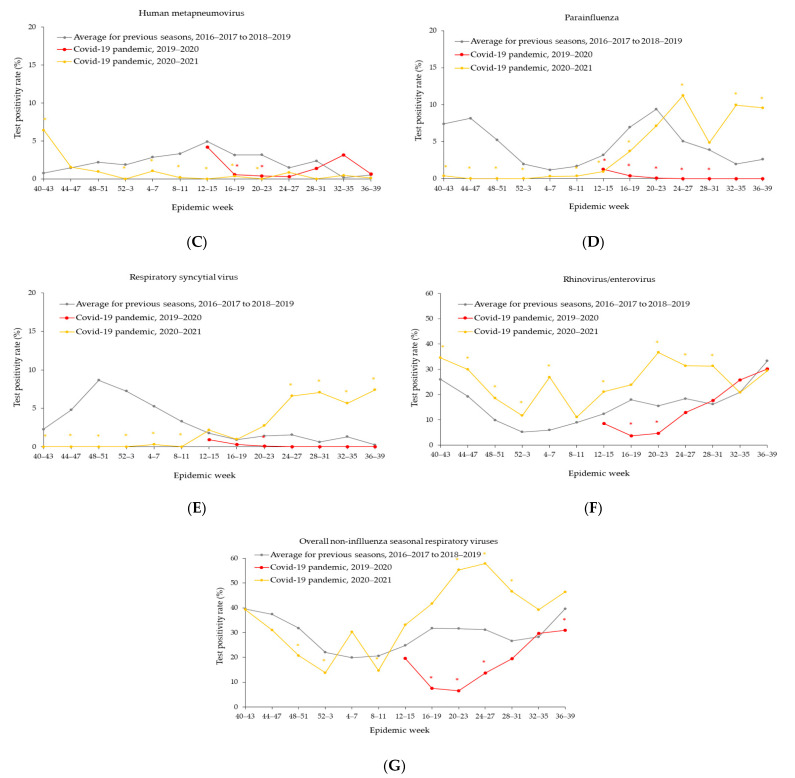
(**A**–**G**) Four-week average positivity rate of non-influenza seasonal respiratory viruses. Epidemic week is the week of the epidemiologic year for which there is the National Notifiable Diseases Surveillance System (NNDSS) disease report, the United States. For the 2019–2020 season, data started from week 12–15. There are 53 weeks exceptionally in the 2020–2021 season; the average positivity rate in week 52–3 was the average of five weeks (52, 53, 1, 2, and 3). *: *p* < 0.05, comparison between averages for three previous seasons, 2016–2017 through 2018–2019 and the COVID-19 pandemic (2019–2020); *: *p* < 0.05; comparison between averages for three previous seasons, 2016–2017 through 2018–2019 and the COVID-19 pandemic (2020–2021).

The four-week average positivity rates for non-influenza seasonal respiratory viruses are shown in Figure 3A,G. During the 2019–2020 season, starting in week 12–15 (12 March –17 April 2020), the four-week average positivity rates for all non-influenza seasonal respiratory viruses (excluding rhinovirus/enterovirus) dramatically decreased or were sustained at very low levels compared to those in the pre-pandemic. As for rhinovirus/enterovirus, the four-week average positivity rates decreased from 8.5% in week 12–15 (21 March–17 April 2020) to 4.7% in week 20–23 (16 May–12 June 2020), then started to increase to 12.9% by week 24–27 (13 June–10 July 2020), and remained at the pre-pandemic levels through the end of the 2019–2020 season. During the 2020–2021 season, there were persistently low circulations of adenovirus and human metapneumovirus, excepting for an uptick (6.4%; *p* < 0.05) observed for human metapneumovirus at the beginning of the 2020–2021 season (Figure 3A,C). In contrast, the activities of seasonal coronavirus, parainfluenza, and RSV continued to be at very low levels following the end of the 2019–2020 season, but started to increase in the 2020–2021 season around week 8–11 (27 February–26 March 2021), then reached pre-pandemic levels or even higher (Figure 3B,D,E). Throughout the entire 2020–2021 season, the four-week averages of positivity rates of rhinovirus/enterovirus were mostly higher (*p* < 0.05) than those in the pre-pandemic, correspondingly (Figure 3F). During the 2020–2021 season, the activity of overall non-influenza seasonal respiratory viruses started to rise in week 8–11 (27 February–26 March 2021), then increased to 41.7% by week 16–19 (24 April–21 May 2021), and remained at high levels through the end of the 2020–2021 season, peaking at a four-week average positivity rate of 57.9% in week 24–27 (19 June–16 July 2021) (Figure 3G).

## 4. Discussion

The emergence of the COVID-19 pandemic triggered a lot of interest from scientists on understanding the relationships between SARS-CoV-2 infection and infections with other respiratory viruses and on predicting what influenza viruses would emerge following the COVID-19 pandemic [16,17]. In the present study, we report the impact of the COVID-19 pandemic and its associated NPIs on laboratory-confirmed detections of influenza and other seasonal respiratory viruses among the Department of Defense personnel in the United States. It is evident from the analysis that the patterns of seasonal respiratory viruses have substantially changed during the COVID-19 pandemic.

The seasonal cycle of respiratory viral infections has been widely recognized [18]. Nevertheless, after implementing NPIs in response to the first wave of the COVID-19 pandemic in the United States, the 2019–2020 influenza season terminated earlier, and most of the seasonal respiratory viruses were suppressed in the early phase of the pandemic in 2020. Consistently, previous studies [19,20] observed a dramatic decrease in seasonal respiratory viruses in the similar period of time. Moreover, it was reported that following the end of the 2019–2020 season, the circulation of influenza and most of the other seasonal respiratory viruses continued to be very low through early 2021 [4,21,22]. In contrast, rhinovirus/enterovirus behaved differently. Studies in other countries [9,19,23] have similar observations showing a rhinovirus resurgence once preventive measures were being loosened during the pandemic in 2020. In addition, Groves et al. [21] observed the continued detection of rhinovirus/enterovirus in Canada through early 2021. The reason causing the difference in rhinovirus circulation in comparison with other seasonal respiratory viruses during the COVID-19 pandemic is unclear. Rhinovirus is a structurally non-enveloped virus, circulating year-round throughout the world [24]. A possible explanation for this phenomenon is non-enveloped rhinovirus may be less susceptible to preventive measures such as hand washing and surface cleaning [25]. In addition, the transmission of rhinovirus occurs by droplets or aerosols, or through direct contact or a fomite [24,26]. Rhinovirus may have more preferable transmission routes; thus, more stringent NPIs are likely required to prevent it from spreading, compared to other seasonal respiratory viruses.

What is notable in the present study is typical winter viruses, such as seasonal coronavirus and RSV [18], returned out of season in 2021 (Figure 1E,H and Figure 3B,E). Moreover, infections with parainfluenza viruses, which were at very low levels for around 12 months since the start of the COVID-19 pandemic, have started to surge in the spring of 2021 in the settings of relaxed NPIs (Figure 1G and Figure 3D). Off-season increased RSV activity has been observed in Australia [27,28] in 2020. In addition, RSV activity has increased in many parts of the United States in the spring of 2021 [4,12,29]. It is unknown for certain what caused the respiratory viruses like RSV to surge out of season. It is possible that RSVs have almost completely disappeared for a year since NPIs were put in place during the pandemic resulting in waning immunity. Then, the reduced immunity led to a potentially large part of the population being more susceptible to RSV infection. Further, the relaxation of NPIs in the United States in the spring of 2021 gave the virus an opportunity to spike. The finding that the easing of NPIs has led to the reemergence of seasonal respiratory viruses highlights the effectiveness of NPIs in preventing the spread of respiratory viruses [29]. Interestingly, we observed a sudden increased activity of influenza A(H3N2) during the last three weeks of the 2020–2021 season (Figure 1B). All of the influenza A(H3N2) infections occurred in the same location in the eastern US. It is unclear whether the influenza A(H3N2) infections indicate that the spreading of influenza is under way. However, it was warned that influenza in the winter 2021/2022 could be around twice the magnitude of a ‘normal’ year [30].

The present study is subject to several limitations. First, it relies on the DoDGRS program for data acquisition. The specimens were collected through routine outpatient clinical care from the sentinel and participating sites in the DoDGRS program. The possibility of potential case selection bias could not be ruled out in the pre-pandemic period or during the COVID-19 pandemic. Second, potential changes in specimen sampling eligibility driven by the COVID-19 pandemic might occur, which could result in a difference in the detection of influenza and other seasonal respiratory viruses compared to those in the pre-pandemic. Third, the healthcare-seeking behavior might have been changed related to SARS-CoV-2 concerns. Patients with respiratory viral infections appeared more likely to take SARS-CoV-2 testing rather than testing for influenza and other seasonal respiratory viruses as before. This might partially explain the lower number of influenza and other seasonal respiratory viruses’ testing during the 2020–2021 respiratory season (Table 2). Another contributing factor could be that the laboratories in sentinel or participating sites might have been overburdened by the large scale of testing for SARS-CoV-2, consequently leading to reduced testing for seasonal respiratory viruses [21].

## 5. Conclusions

In conclusion, the present study demonstrates that the COVID-19 pandemic and its associated NPIs have exerted tremendous impacts on the circulations of influenza and other seasonal respiratory viruses. The findings, based on the analysis of a database from the DoDGRS program, make significant additions to the growing evidence that NPIs may be an effective public health tool in reducing the spreading of seasonal respiratory viruses. The reason for the dramatic decrease in transmissions of influenza and other seasonal respiratory viruses during the COVID-19 pandemic remains unclear. In addition, little is known as to why some respiratory viruses (seasonal coronavirus, RSV) rebounded out of season following the easing of NPIs. Further study is needed to explore and thus have a better understanding of the underlying mechanisms for altering circulation patterns of seasonal respiratory viruses. With the ongoing COVID-19 pandemic, it is important to evaluate transmission dynamics and risks of influenza and other seasonal respiratory viruses in subsequent respiratory seasons. Indeed, assessing the exact prevalence of respiratory viruses in the real world would undoubtedly provide valuable information, and help guide the design of preventive measures in mitigating seasonal respiratory virus epidemics.

## Figures and Tables

**Table 1:** Influenza and other seasonal respiratory viruses detected over five respiratory seasons from 2016–2017 to 2020–2021.

		Number of Specimens in Respiratory Virus Season ^b^				
Pathogen ^a^	Overall	2016–2017	2017–2018	2018–2019	2019–2020	2020–2021
Influenza A(H1N1)pdm09						
Single infection	3430	28	393	835	2173	1
Co-infection	339	ND	41	57	241	ND
Influenza A(H3N2)						
Single infection	4286	1252	1426	1483	72	53
Co-infection	338	2	161	153	15	7
Influenza A/Not subtyped						
Single infection	1465	ND	6	1426	33	ND
Co-infection	10	ND	1	3	6	ND
Influenza B						
Single infection	2378	424	713	125	1113	3
Co-infection	1243	1	258	40	944	ND
Dual influenza	47	4	17	3	23	ND
Non-influenza virus						
Adenovirus	634	75	107	235	197	20
Seasonal coronavirus	2189	110	503	633	763	180
Human metapneumovirus	1338	91	320	203	681	43
Parainfluenza	1208	195	187	321	327	178
Respiratory syncytial virus	1649	175	398	378	573	125
Rhinovirus/Enterovirus	6687	344	992	1214	2692	1445
Co-infection	1623	131	366	392	599	135
Other pathogen	491	42	92	110	229	18
No pathogen detected	33,121	2310	2945	11,440	12,511	3915

^a^ Co-infection: co-infection with one or more non-influenza pathogens. ^b^ ND: not detected.

**Table 2:** Characteristics of seasonal pattern of influenza activity over five respiratory seasons from 2016–2017 to 2020–2021.

	Influenza Season ^b^				
Variable ^a^	2016–2017	2017–2018	2018–2019	2019–2020	2020–2021
Start of epidemic period					
Epidemic week	48	46	52	46	/
Date	3–9 December 2016	18–24 November 2017	29 December 2018–4 January 2019	16–22 November 2019	/
End of epidemic period					
Epidemic week	19	17	16	11	/
Date	13–19 May 2017	28 April–4 May 2018	20–26 April 2019	14–20 March 2020	/
Duration of epidemic period, week	24	24	17	18	/
Peak rate of test positivity					
Level, %	49.6	53.7	43.7	47.5	/
Epidemic week	8	52	7	7	/
Date	25 February– 3 March 2017	30 December 2017– 5 January 2018	16–22 February 2019	15–21 February 2020	/

^a^ Epidemic week is the week of the epidemiologic year for which there is the National Notifiable Diseases Surveillance System (NNDSS) disease report, the United States. ^b^ The 2020–2021 influenza season did not occur.

## Data Availability

All data are presented in this article. For further information, contact the corresponding author.
